# GUEST EDITORIAL: Theranostics in breast cancer—a vision

**DOI:** 10.1007/s00432-023-05242-8

**Published:** 2023-08-07

**Authors:** Hans-Jürgen Biersack, Klaus Höffken

**Affiliations:** 1 Department of Nuclear Medicine, University Hospital Bonn, Venusberg-Campus 1, Gebäude A21, 53127 Bonn, Germany; 2 Journal of Cancer Research and Clinical Oncology, Welserstr. 21, 10777 Berlin, Germany

Nuclear medicine offers imaging procedures for breast cancer since over 35 years now, including the estrogen receptor targeting. First results of I-123 estradiol were published in 1990 shortly after PET imaging of estrogen receptors had been reported. F-18 FES (fluoro-17beta-estradiol) has also successfully been used in ER + breast cancer under treatment with an ER degrader.

Other radiopharmaceuticals—not targeting the ER receptor—used for breast cancer imaging comprised F-18 FDG or Tc-99 m MIBI. Another approach required Herceptin targeting of HER2-expressing tumors Other studies used HER2-binding Ga-68_ABY-025 or Tc-99 m DARPin.

All these radionuclide-based imaging procedures were useful to detect breast cancer, but not for fighting the malignant cells.

The combination of imaging and treating malignant tumors called “Thera(g)nostics” has been successfully applied in somatostatin-positive neuroendocrine tumors where it was proven that the median progression-free survival in patients with NET of duodenum and pancreas was significantly extended compared to other therapeutic procedures (Kipnis et al. 2021).

Since 2 years now, thera(g)nostics play a significant role in prostate cancer: It is a step-by-step approach to diagnosis and therapy for mCRPC (castration-resistant prostate cancer). In 2022, the results of the VISION trial in mCRPC were published in JAMA (Sartor et al. 2021), comprising the data of 831 patients (out of 1179 screened). The progression-free survival could be extended from 3.4 to 8.7 months and overall survival from 11.3 to 15.3 months. On the basis of these data, Lutetium-177-PSMA-617 was accepted for clinical use by FDA and EMEA.

These data make evident, that a similar success may be expected in breast cancer, if the appropriate radiopharmaceuticals become available for precision oncology.

For this purpose, NanoMab offers camelide nanobodies like anti-HER2 (Altunay et al. 2023). Currently, nanobodies against the ER receptor with its four epitopes are tested. In ER receptor targeting for diagnosis and therapy—similar to somatostatin receptor targeting—the potential of agonists and antagonists has to be evaluated. The appropriate therapeutic agent has to be checked for the intratumoral residence time, as the half-life of the isotope has to be adapted (long half-life in long residence time and vice versa, e.g., Lu-177 vs. Re-188). Therapy with ER receptor agents goes far beyond the properties needed for imaging. Coactivator peptides have been proposed for differential stabilizing effects on the binding of estrogens and antiestrogens which may lead to alteration of the residence time of the radiopharmaceutical.

For practicing oncologists, availability of radiolabeled antiestrogens or antibodies against EGRF or Ki67 may become a valuable alternative in treating in situ relapses or metastases to lymph nodes or organs if detected by radio-PET-CT. It may be even more effective than antibodies that (only) block the receptors and inhibit growth in that the radiolabeled antibodies will destroy the tumor cell. In addition, the radiolabeled antibodies may be used to detect relapses during follow-up after primary treatment of breast cancer.

Clinical studies are urgently awaited to make proof of principles and clinical efficacy.

In summary, it can be envisaged that theranostics of breast cancer will become the turning point in precision treatment of breast cancer.
